# The effects of amisulpride on five dimensions of psychopathology in patients with schizophrenia: a prospective open- label study

**DOI:** 10.1186/1471-244X-5-22

**Published:** 2005-05-03

**Authors:** Miguel Herrera-Estrella, Rogelio Apiquian, Ana Fresan, Isabel Sanchez-Torres

**Affiliations:** 1 Fray Bernardino Alvarez Psychiatric Hospital, Mexico City, Mexico; 2 Psychiatry Department, National Institute of Neurology and Neurosurgery, Mexico City, Mexico; 3 Clinical Research Division, National Institute of Psychiatry, Mexico City, Mexico; 4 Carracci Medical Group, Mexico City, Mexico; 5 Sanofi- Synthelabo, Mexico City, Mexico

## Abstract

**Background:**

The efficacy of antipsychotics can be evaluated using the dimensional models of schizophrenic symptoms. The D_2_/D_3_-selective antagonist amisulpride has shown similar efficacy and tolerability to other atypical antipsychotics. The aim of the present study was to determine the efficacy of amisulpride on the dimensional model of schizophrenic symptoms and tolerability in latin schizophrenic patients.

**Method:**

Eighty schizophrenic patients were enrolled and 70 completed a prospective open-label 3-month study with amisulpride. The schizophrenic symptoms, psychosocial functioning and side-effects were evaluated with standardized scales.

**Results:**

The patients showed significant improvement in the five dimensions evaluated. Amisulpride (median final dose 357.1 mg/d) was well-tolerated without treatment-emergent extrapyramidal side-effects.

**Conclusion:**

Amisulpride showed efficacy on different psychopathological dimensions and was well tolerated, leading to consider this drug a first line choice for the treatment of schizophrenia.

## Background

The treatment of schizophrenia has shown important improvements since the introduction of new antipsychotics [1]. These drugs, also called atypical or second generation antipsychotics (SGAs) bring the possibility of a better quality of life for patients affected with schizophrenia, because they have been associated with a better efficacy over negative symptoms, probably less cognitive impairment and lower probability of extrapyramidal symptoms (EPS) [2], which is one of their main advantages.

The SGAs increase the release of dopamine in the prefrontal cortex and the hippocampus [3]. This effect of SGAs is critical to improve negative symptoms and cognition, and to decrease the EPS. The principal hypothesis of their mechanism of action has been attributed to the antagonism of 5-HT_2A _receptors coupled to weaker antagonism of dopamine D_2 _receptors [4,5]. Their effect as 5-HT_1A _receptor agonist has also been suggested to contribute to an atypical antipsychotic profile [6].

Nevertheless, amisulpride represents an important contrast on the theory of the 5HT_2A _receptor antagonism. Amisulpride, is a benzamide with high affinity for dopamine D_2 _and D_3 _receptors without affinity for serotonin, muscarinic or alpha-adrenergic receptors [7]. Amisulpride also shows selectivity for dopamine receptors in limbic and hippocampal structures, rather than striatal region [8].

At low doses (100–300 mg/d), amisulpride binds preferentially on D_2_/D_3 _presynaptic autoreceptors [8,9], increasing dopaminergic transmission in the prefrontal cortex, which is believed to be associated with improvement of primary negative symptoms. Doses between 400–800 mg/d result in antagonism of postsynaptic dopamine receptors [8], leading to an improvement of positive symptoms of schizophrenia with less EPS development.

The limbic selectivity of amisulpride is similar to the observed with clozapine, and is secondary to its high affinity for D_3 _receptors and the short isoform of the D_2 _receptor, which are highly distributed in these regions [10]. This selectivity has been documented in animal models [7]. In addition, PET studies have shown that receptor D_2 _occupancy in striatal regions is around 14% when amisulpride is prescribed at doses between 50–100 mg/d [11]. A decreased amisulpride plasma concentration induces a low percentage of occupancy in striatal and increased occupancy in limbic regions [12].

The improvement of negative symptoms has been documented in clinical studies with doses between 50–100 mg/d [13,14]. Until now, amisulpride is the only antipsychotic that has shown scientific evidence of its efficacy over the primary negative symptoms of schizophrenia [15-18]. Several clinical trials have shown that amisulpride has similar efficacy and better tolerability in comparison to haloperidol and flupentixol [19-22] as well as similar efficacy and safety when compared to olanzapine and risperidone [23-27]. Additionally, amisulpride has shown a positive effect over depressive symptoms [28,29] and the cognitive impairment [30,31] related to schizophrenia.

These data support that amisulpride is also an 'atypical' antipsychotic despite having a different receptor-affinity profile. However, there is a lack of studies about the efficacy and tolerability of amisulpride in latin populations. We decided to perform a 3-month open trial to determine these parameters on a sample of Mexican patients, using the five-factor model of schizophrenic psychopathology, a previously determined useful strategy for the evaluation of drug efficacy [32].

## Method

Subjects were consecutively recruited from the inpatient and outpatient services from 7 centers in Mexico between August 2001 and January 2003. The study was conducted in accordance with the Good Clinical Practices and the Declaration of Helsinki. The study protocol was approved by an Institutional Review Board and Ethics Committee at each center. Written informed consent was obtained after the procedures had been fully and detailed explained to patients.

The sample comprised men and nonpregnant, nonlactating women aged 18 to 65 years who met diagnostic criteria of schizophrenia (as per DSM-IV), including patients on their first-episode psychosis (defined as their first psychiatric admission due to psychosis with a maximum duration of untreated illness of 5 years), patients with acute psychotic symptoms (defined by baseline PANSS total score of at least 60 with a minimum score of 4 on at least 2 items of the positive subscale) due to antipsychotic treatment noncompliance and patients requiring treatment switch due to lack of efficacy (positive and/or negative symptom persistence) or presence of adverse events. Patients were excluded if they had history of bipolar disorder, high risk for suicide or agitation/violence, concomitant medical or neurological illness (as per review of systems, and general physical examination) and DSM-IV defined current substance abuse or a history of substance dependence in the last six months.

### Assessments

Diagnoses were based on the Structured Clinical Interview for DSM-IV Axis I Disorders (SCID-I) [33] according to DSM-IV criteria. Efficacy was rated using the dimensional models of schizophrenic symptoms, based on confirmatory 5 factor analysis (positive, negative, cognitive, excitement and depression/anxiety factors) of the Positive and Negative Syndrome Scale (PANSS) (each item rated from 1 to 7) in Mexican population [34], the Clinical Global Impression Scale (severity and improvement subscales rated from 0 to 7) [35] and the Calgary Depression Scale for Schizophrenia (CDSS) [36] for depressive symptoms. The efficacy of amisulpride was examined on other dimensions of schizophrenia, such as psychosocial functioning, with the Psychosocial Aptitude Rating Scale (PARS) (10 items rated from 0 to 10, where a total score of "0" denotes the least healthy end of the functioning range, and "100" the healthiest end) [37]. Tolerability was assessed with the Barnes Akathisia Scale (BAS) for akathisia [38], the Simpson Angus Scale (SAS) for EPS [39] and the Abnormal Involuntary Movements Scale (AIMS) for dyskinesia [40]. For each patient, the same investigator rated these measures at baseline, day 15, day 30 and day 90.

### Treatment

This was an open label study using flexible doses (100 mg/d – 1200 mg/d) of amisulpride. Patients with predominantly negative symptoms, defined as a greater score on the negative than on the positive subscale in the PANSS, a negative subscale score >20, at least moderate global negative symptom score (items score as 4) and absence of depression [41] received an initial dose of 100 mg/d – 300 mg/d, while patients with predominantly positive symptoms (defined as a greater score on the positive than on the negative subscale in the PANSS) [41] received an initial dose ≥400 mg/d. During the 3-month treatment phase, the drug doses were adjusted based on clinical considerations, treatment response and side effects. Patients with lack of clinical response and/or presence of adverse events with the antipsychotic they were taking before were switched to amisulpride tapering off the previous antipsychotic agent and gradually titrating amisulpride to the established therapeutic dose. Biperiden (2–12 mg/d) was prescribed for the treatment of EPS, lorazepam (0.5–4 mg/d) for anxiety and akathisia, and antidepressant medication was allowed for the treatment of depressive symptoms.

### Analysis

Demographic and clinical characteristics description was done with frequencies and percentages for categorical variables and with means and standard deviations (S.D.) for continuous variables. The effect of treatment was assessed using repeated measures ANOVA for continuous contrasts. Where appropriate, paired t-test (two tailed) were conducted to examine the differences. For the analysis of patients with predominantly positive symptoms and predominantly negative symptoms, changes from baseline to endpoint in PANSS dimensions and the PARS score were examined and compared using an analysis of covariance (ANCOVA) model adjusted for baseline scores. The improvement criteria a priori specified in the study required a ≥ 20% decrease from baseline in PANSS total score to study endpoint. However, we also examined the results with ≥ 30% decrease from baseline in PANSS total score as the criterion for response, as the latter may be more clinically meaningful. Chi square test (x^2^) was used for the comparison of improvement and response criteria between patients with predominantly positive symptoms and predominantly negative symptoms. The significance level for tests was established at p = 0.01 to decrease the Type 1 error as a consequence of multiple comparisons.

## Results

A total of 80 patients were included, their demographic and clinical characteristics are described in table 1. The 3-month treatment phase was completed by 70 (87.5%) patients. The most frequent reason for premature withdrawal was the lack of clinical response (n = 6). The other reasons for discontinuation from the study were the lost to follow-up (n = 3), serious adverse events (severe EPS in one patient and overdosing in one patient) and non-compliance (n = 1). Non-compliance was determined when patients discontinued their treatment for a period longer than 1 week. Only three patients with first-episode were discontinued from the study. The data from these 70 patients remaining in the study is reported.

**Table 1:** Demographic and clinical characteristics of the sample

	n (%)
**Gender**	
Male	60 (75)
Female	20 (25)

**Schizophrenia subtype**	
Paranoid	40 (50)
Undifferentiated	37 (46.2)
Disorganized	2 (2.5)
Residual	1 (1.3)

Inpatients	20 (25)
Outpatients	60 (75)

**Switched from previous antipsychotic**	48 (60)
Noncompliance	20 (25)
First Episode	12 (15)

	Mean (S.D.)

Age	33.1 (10.3)

Age at onset	22.6 (6.7)

Length of illness (yr.)	10.5 (8.2)

PANSS total score	90.9 (23.8)

The mean initial dose of amisulpride was 335.7 mg/day (S.D. = 147.4, median 350 mg/day) and the mean dose at the end of the study was 357.1 mg/day (S.D. = 159.3, median 400 mg/day). The amisulpride doses were not different between first episode and chronic patients.

### Efficacy data

Treatment with amisulpride was associated with a highly significant improvement on the five factors and on the total score of the PANSS. The improvement was observed from the end of the second week with a substantial reduction in the severity of the symptoms, patients reached a high level of improvement evaluated with CGI-I and this level was maintained at the end of third month. The depressive symptoms evaluated with CDSS were mild to moderate at baseline, the CDSS total score decreased significantly through the study (Table 2).

**Table 2:** Effects of treatment on PANSS factors, depressive symptoms and the clinical global impression.

	**Baseline**	**Week 2**	**Week 4**	**Week 12**	**Statistics**	**p value**
**PANSS Factors**						
Positive	25.7 (8.6)	18.2 (6.6)	14.7 (7.1)	13 (6.6)	F(3,77) = 33	<0.001
Negative	26.8 (8.3)	19.3 (7.1)	16.3 (7.3)	13.8 (6.4)	F(3,77) = 41.1	<0.001
Cognitive	19.8 (6.2)	14 (5.2)	12.8 (5.2)	11.2 (5)	F(3,77) = 24.4	<0.001
Excitement	9.4 (4.1)	6.1 (2.8)	5.6 (3)	5.2 (2)	F(3,77) = 17.6	<0.001
Depression/Anxiety	9.7 (4.3)	7.3 (2.9)	6.4 (3)	5.4 (2.6)	F(3,77) = 21.6	<0.001
Total Score	91.5 (23.8)	78.4(16.9)	56 (21.9)	48.8 (20.1)	F(3,77) = 43.6	<0.001

**CDSS**	3.9 (4.9)	2.4 (3)	1.7 (2.9)	1 (2.7)	F(3,77) = 9.6	<0.001

**CGI**						
Severity	4.7 (1.1)	3.4 (1.1)	2.8 (1.2)	2.3 (1.4)	F(3,77) = 54.2	<0.001
Improvement		2.5 (0.9)	2.2 (1)	2.3 (1.4)	F(2,78) = 2.1	0.12

Mean (S.D.)

PANSS, Positive and negative syndrome scale

CDSS, Total score of the Calgary depression scale for schizophrenia

CGI, Clinical global impression scale

Amisulpride treatment also improved the social functioning measured with the PARS (baseline = 47.6, S.D. = 18.6 vs 3-month = 73.9, S.D. = 17.2, t = 10.3, df = 69, p < 0.001) and none was an inpatient at the end of the study.

A higher frequency of patients with predominantly positive symptoms was found (n = 40, 57%). All negative symptom subjects were classified as having the undifferentiated subtype of schizophrenia according to DSM-IV criteria. The mean final doses were 343 mg/day (S.D. = 143.1, median 350 mg/day) for the predominantly negative group and 392.5 mg/day (S.D. = 183.1, median 400 mg/day) for the predominantly positive subjects. Amisulpride was associated to similar improvement in all the five-factors of the PANSS. The mean baseline and changes from baseline to endpoint in PANSS dimensions scores for both groups are shown in Table 3. The predominantly positive patients showed a lower psychosocial functioning at baseline when compared to the predominantly negative group (t = 3.58, df 68, p = 0.001), however, no statistically significant differences emerged between groups at the end of the study (predominantly negative = 72.5 SD = 20.19; predominantly positive = 75.07 SD = 14.74, t = 0.61, df 68, p = 0.53).

**Table 3:** Mean change from baseline to endpoint in secondary efficacy measures

PANSS Factors	Predominantly Negative (n = 30)		Predominantly Positive (n = 40)		Statistic* Between Groups	Statistic* During the Follow-up
	Mean	SD	Mean	SD		

**Positive**						
Baseline	19.5	6.8	30.4	6.8	F = 1.09, df 1, p = 0.29	F = 79.58, df 1, p < 0.001
Mean Change	-7.2	10.1	-16.7	9.6		

**Negative**						
Baseline	30.8	8.6	23.8	6.8	F = 0.16, df 1, p = 0.68	F = 80.18, df 1, p < 0.001
Mean Change	-16.3	11.2	-10.5	8.1		

**Cognitive**						
Baseline	18.5	6.7	20.7	5.7	F = 0.01, df 1, p = 0.91	F = 111.29, df 1, p < 0.001
Mean Change	-7.1	9.4	-9.6	7.3		

**Excitement**						
Baseline	8.0	4.0	10.4	3.9	F = 2.63, df 1, p = 0.10	F = 304.76, df 1, p < 0.001
Mean Change	-3.1	4.7	-4.9	4.7		

**Depression-Anxiety**						
Baseline	8.9	4.2	10.3	4.2	F = 0.16, df 1, p = 0.68	F = 171.77, df 1, p < 0.001
Mean Change	-3.6	4.9	-4.7	5.1		

**Total score**						
Baseline	85.8	25.1	95.8	22.1	F = 0.05, df 1, p = 0.82	F = 94.80, df 1, p < 0.001
Mean Change	-37.4	35.3	-46.6	28.4		

* Based on analysis of covariance adjusted for baseline score.

### Response rate analysis

The improvement rate in the total sample was 82.9% using the >20% reduction in PANSS total score while response rate was 75.7% using the ≥30% reduction in PANSS total score. Similar improvement rates were found in the predominantly negative patients (73.3%) and in the predominantly positive patients (90.0%) (x^2 ^= 3.35, df 1, p = 0.07). In the same way, similar response rates were found in both groups (predominantly negative 66.7% vs. predominantly positive 82.5%) (x^2 ^= 2.33, df 1, p = 0.12).

### Tolerability data

Amisulpride significantly decreased the EPS and abnormal involuntary movements at the end of the study. There were no significant differences in akathisia's severity during the study, although it remained as mild. There was a trend toward a slight weight increase with the use of amisulpride during the 3-month treatment phase (Table 4).

**Table 4:** Summary of tolerability data during the treatment phase.

	**Baseline**	**Week 2**	**Week 4**	**Week 12**	**Statistics**	**p value**
**SAS**	4.3 (5.9)	2.2 (4)	2.1 (3.7)	2 (3.7)	F (3,77) = 5.1	<0.001
**BAS**	2.2 (3.2)	1.5 (2.8)	1.3 (2.2)	1.3 (2.2)	F (3,77) = 2.2	0.09
**AIMS**	3.8 (5.9)	2.5 (4.8)	2.1 (3.9)	1.8 (4.1)	F (3,77) = 5.0	<0.001
**Weight (kg,)**	70.7 (13.5)	70.5 (12.9)	70.9 (13.2)	71.4 (13.2)	F (3,77) = 2.3	0.08

Mean (S.D.)

SAS, Total score of the Simpson Angus scale

BAS, Total Score of the Barnes akathisia scale

AIMS, Total score of the abnormal involuntary movements scale

A total of 10 patients (12.5%) required the use of biperiden (4.0 mg/day, S.D. = 3.1) for EPS control at baseline, only 2 patients (2.8%) continued on anticholinergic medication (1.5 mg/day, S.D. = 0.70) at the end of the study. In addition 2 patients (2.5%) required antidepressants (paroxetine and reboxetine, respectively) at the beginning of the study but none at the end of the study. Sixteen 16 patients (20%) required lorazepam for anxiety or insomnia at baseline, but only 3 (4.2%) required this drug at the end of the study.

Akathisia was the main adverse event reported during the study (n = 4), followed by headache (n = 1), insomnia (n = 1) and amenorrhea (n = 1). All side effects were reported as mild.

## Discussion

The factor-analysis studies of the PANSS produced five factors (positive, negative, cognitive, excitability/hostility and depression/anxiety), this solution fits better to the multidimensional model described for the psychopathology of schizophrenia [42-44]. Our results demonstrated a substantial benefit with amisulpride on the five dimensions of psychopathology based on confirmatory factor analysis of the PANSS in Mexican schizophrenic patients. This confirms the properties of amisulpride as an atypical antipsychotic given its efficacy and low frequency of EPS showed in this study. The findings of this study resemble previous reports of the efficacy of amisulpride among the dimensions obtained from factor analysis of BPRS [22,28,29].

Amisulpride also improved the psychosocial functioning of the patients and was associated with discharge from the hospital in subjects who were initially inpatients. These two variables could be associated with the drug's efficacy, better tolerability, or both. It has been observed that atypical antipsychotics improve the functioning and reduce the global negative symptoms; amisulpride has shown similar efficacy to the reported of risperidone [23,26] and olanzapine [24,27] in the treatment of these symptoms.

Similarly to previous reports, amisulpride improved the depressive [22,29] and cognitive symptoms [30,31]. This finding also increase the evidence of the atypical properties of amisulpride, despite its action as D_2_/D_3 _antagonist without affinity with other neurotransmitter systems.

In the current study, amisulpride produced substantial and significant reductions in psychopathology during the follow-up both in patients classified with predominantly negative or predominantly positive symptoms, without statistically significant difference between the groups. This finding could be explained by the dual pharmacodynamic effect of amisulpride [7-9]. Although both improvement and clinical response were observed in the groups according to predominant symptoms; patients with predominantly positive symptoms showed a trend to improve in higher rates.

The mean dose used in this study was lower than the doses reported in other studies which included patients with acute exacerbation of positive symptoms (600 mg/day) [45], given that the present sample included patients with predominantly negative symptoms, which have been reported to respond to lower doses of amisulpride. However, the final doses for patients with predominantly negative symptoms and predominantly positive symptoms did not differ, probably because some patients from the first group showed an increase on the severity of positive symptoms during the follow up. The most frequently prescribed dose was 400 mg/day for the treatment of patients with acute symptoms and first episode of psychosis.

Amisulpride was well tolerated, the need for concomitant medication was reduced at the end of the study and the reported adverse events were mild. The total weight increase was 0.7 kg during the 3-month treatment phase. This increase was similar to the reported in other studies [46] and lower to the 1.4 kg increase reported in previous meta-analysis [47]. This finding could be explained by the fact that in 20 patients (28.6%) a mean 2.1 kg of weight decrease was registered. Indeed, it has been demonstrated in randomized 6 months follow-up studies that amisulpride is associated with less weight gain than risperidone or olanzapine [25,27].

The observed decrease on the abnormal involuntary movements at the end of the study could also be explained by the treatment switch. Although only one patient reported an adverse event associated with hyperprolactinemia, this adverse event could not be rejected since in this study prolactin levels were not regularly measured. Amisulpride tended to increase prolactin levels when compared with other atypical antipsychotics [48,49]. Some studies reported few adverse events related with hiperprolactinemia [50], this is probably due to the decrease of prolactin levels with the long term treatment [51].

The findings of this study were similar to those observed in other international clinical trials with amisulpride, but should be considered with caution since one of its limitations was the open label design with a short term follow-up. Another limitation was that the outpatients outnumbered the inpatients, since the high cost of treatment in Mexico does not permit to afford the inpatient services. This sample distribution could not allow the results to be generalized to other countries where subjects with moderate or severe symptoms are treated as inpatients. Further studies should include adequate instruments for the assessment of cognitive symptoms as well as laboratory measures of prolactin, glucose and lipids levels.

## Conclusion

The efficacy and tolerability profile of amisulpride in a Mexican population of schizophrenic patients is similar to that reported with other second generation antipsychotics, leading to consider this drug as first line for the treatment of schizophrenia.

## Competing interests

This study was supported by Sanofi-Synthelabo. MHE has been member of advisory board of Bristol-Myers Squibb and has received funding from Pfizer Inc, Eli Lilly, Bristol-Myers Squibb and Sanofi-Synthelabo and he has served on the speakers bureau of Sanofi-Synthelabo, Bristol-Myers Squibb, Janssen Cilag and AstraZeneca Pharmaceuticals. RA has been a consultant and member of advisory board of Bristol-Myers Squibb and has received funding from Bristol-Myers Squibb, Sanofi-Synthelabo, Janssen Cilag and Theodore and Vada Stanley Foundation; he has served on the speakers bureau of Sanofi-Synthelabo, Bristol-Myers Squibb, and Janssen Cilag. IST is affiliated to Sanofi-Synthelabo.

## Authors' contributions

RA, MHE and IST drafted the manuscript. AF performed the statistical analysis. RA, MHE and IST participated in the study design and coordination. All authors read and approved the final manuscript.

## Pre-publication history

The pre-publication history for this paper can be accessed here:


